# Multiterritory Acute Ischemic Stroke with Bilateral Middle Cerebellar Peduncle T2/FLAIR Hyperintensities in the Setting of a Fatal Systemic Arterial Thrombotic Process: A Case Report

**DOI:** 10.1016/j.radcr.2026.08.046

**Published:** 2026-09-15

**Authors:** Kessé Marc-Antoine Brou, Seydou Traoré, N’guessan Judicaël Ahoury, Niamkey Aristide Aka, Kouassi Paul N’zi

**Affiliations:** a Department of Anatomy, Felix Houphouët-Boigny University, Abidjan, Côte d'Ivoire; b Department of Radiology, Heart Institute Abidjan, Abidjan, Côte d'Ivoire; c Department of Radiology, Felix Houphouët-Boigny University, Abidjan, Côte d'Ivoire

**Keywords:** Ischemic stroke, Middle cerebellar peduncle, MRI, Systemic arteriopathy, Antiphospholipid syndrome

## Abstract

Ischemic stroke under 55 years presents a distinct etiological challenge with higher prevalence of non-atherosclerotic causes. We report a fatal case of multiterritory acute cerebral infarction with bilateral middle cerebellar peduncle (MCP) T2/FLAIR hyperintensities in a 50-year-old woman, pursuing two educational objectives: (1) provide a radiological framework for interpreting bilateral MCP T2/FLAIR hyperintensities without diffusion restriction in the context of basilar occlusion; and (2) illustrate a diagnostic approach to multiterritory arterial thrombosis concurrent with systemic infection. A 50-year-old Ivorian woman with hypertension, diabetes, and severe LEAD complicated by right lower limb trophic ulcers and local infection was admitted for sudden-onset right hemiplegia (NIHSS 9) occurring one week earlier. Brain MRI showed acute infarcts in the left corona radiata and right paramedian pons, Fazekas grade 2 leukoaraiosis, a chronic callosal lesion, and symmetric bilateral MCP T2/FLAIR hyperintensities without diffusion restriction or enhancement. MR angiography revealed left M1 occlusion and absent basilar artery flow. Transthoracic echocardiography excluded intracavitary thrombus. Laboratory tests showed leukocytosis (45,190/mm³, 90.7% neutrophils), anemia (Hb 8.1 g/dL), elevated CRP (192 mg/L), and hyperuricemia. The patient died three weeks after admission from sepsis and multiorgan failure following above-knee amputation. Antiphospholipid and thrombophilia work-up could not be completed. Bilateral MCP T2/FLAIR hyperintensities without diffusion restriction or enhancement, in the setting of basilar occlusion and longstanding leukoaraiosis, are most consistent with chronic ischemic gliosis. Multiterritory arterial thrombosis concurrent with active systemic infection should prompt immediate parallel evaluation for thromboinflammatory conditions, including urgent antiphospholipid antibody testing, before irreversible clinical decisions are made.

## Introduction

Ischemic stroke in adults under 55 years of age presents a diagnostic challenge distinct from that of older patients. Non-atherosclerotic etiologies — including cardio-embolism, arterial dissection, thrombophilia, and vasculitis — account for a disproportionately large share of cases in this age group [1,2]. The co-occurrence of simultaneous acute infarcts across multiple arterial territories strongly suggests an embolic or systemic thrombotic process and warrants an urgent and comprehensive etiological evaluation [2]. However, the co-occurrence of extensive chronic white matter changes and rare imaging patterns, such as bilateral middle cerebellar peduncle (MCP) T2/FLAIR hyperintensities, may provide crucial additional clues pointing toward an underlying systemic disease [3].

We report a fatal case of a 50-year-old woman with hypertension, diabetes mellitus, and severe lower extremity arterial disease (LEAD) complicated by pre-existing trophic ulcers and local infection, who presented with acute multiterritory cerebral infarction. The case highlights the potential role of infection and systemic inflammation as triggers of a catastrophic thrombotic arteriopathy. Written informed consent was obtained from the patient's family, and the study was approved by the Ethics Committee of the Institut de Cardiologie d'Abidjan.

This case report pursues two explicit educational objectives. First, to provide a structured radiological framework for interpreting bilateral MCP T2/FLAIR hyperintensities without diffusion restriction — a finding with a broad differential diagnosis that carries important clinical implications regardless of whether a final etiological diagnosis is established. Second, to illustrate a systematic diagnostic approach to multiterritory arterial thrombosis in the context of concurrent systemic infection, highlighting a critical clinical decision point: the urgent need to initiate antiphospholipid antibody testing in parallel with imaging, before irreversible clinical decisions are made.

## Case presentation

A 50-year-old Ivorian woman with history of poorly controlled arterial hypertension and type 2 diabetes mellitus was admitted to the emergency department. She had been followed for severe lower extremity arterial disease (LEAD) with right-sided trophic ulcers and local infection of the foot, managed on an outpatient basis. Approximately one week before admission, she had suddenly developed right-sided hemiplegia. The family delayed seeking care because they initially attributed the weakness to her chronic limb condition.

On admission, she was alert. Neurological examination confirmed severe right hemiplegia (muscle strength 2/5 in the right upper and lower limbs) with partial facial asymmetry. No aphasia, cranial nerve palsy, or meningeal signs were noted. NIHSS score was 9. Blood pressure was 165/95 mmHg. Examination of the lower extremities revealed bilateral signs of chronic arterial insufficiency, with right leg trophic ulcers, local erythema, and purulent discharge consistent with soft tissue infection. Pedal pulses were absent bilaterally. Given the one-week delay, acute reperfusion therapy was not indicated.

### Brain MRI findings (Fig. 1)

Brain MRI was performed on a 1.5 Tesla SIEMENS Magnetom Go Top MRI system and included diffusion-weighted imaging (DWI/ADC), T2-weighted, FLAIR, T1-weighted, post-contrast T1-weighted, and time-of-flight (TOF) MR angiography (MRA) sequences.

**Fig. 1:**
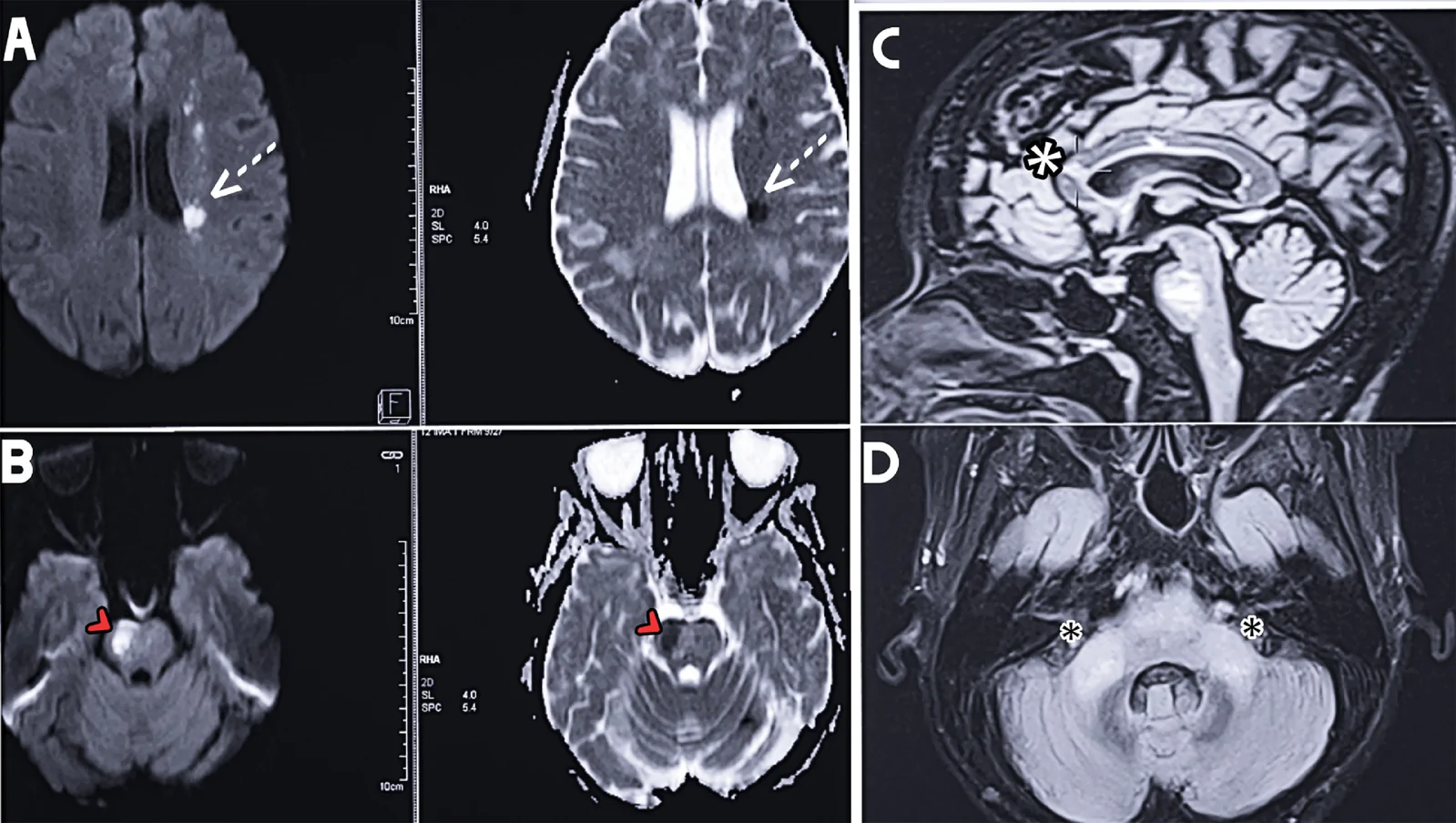
Brain MRI findings. (A) Axial diffusion-weighted image (DWI, left panel) with corresponding apparent diffusion coefficient (ADC) map (right panel) at the level of the corona radiata. The white dashed arrow indicates a focal area of high signal on DWI with corresponding low ADC signal, consistent with diffusion restriction and acute ischemic infarction in the left corona radiata, within the territory of the left middle cerebral artery. (B) Axial DWI (left panel) with corresponding ADC map (right panel) at the level of the pons. The red arrowhead indicates a focal area of diffusion restriction in the right paramedian pons, consistent with a second acute ischemic infarct in the posterior circulation territory, confirming simultaneous multiterritory infarction. (C) Sagittal T2-weighted image at the midline. The asterisk (*) indicates a focal T2 hyperintensity in the genu of the corpus callosum consistent with chronic ischemic gliosis. Note the markedly reduced caliber of the brainstem tegmentum at the level of the pons, reflecting severe atrophic changes in the context of chronic vertebrobasilar occlusive disease. (D) Axial T2-weighted image at the level of the middle cerebellar peduncles (MCPs). Bilateral asterisks (**) indicate symmetric, confluent T2 hyperintensities involving both MCPs, consistent with chronic ischemic gliosis secondary to severe vertebrobasilar occlusive disease, rather than acute infarction.

**Acute/subacute ischemic lesions:** A focal area of diffusion restriction (high signal on DWI, corresponding low ADC) was identified in the left corona radiata. A second similar focus was present in the paramedian region of the right pons (Fig. 1A, 1B). The persistence of diffusion restriction at seven days post-ictus is consistent with subacute infarction in both vascular territories.

**Fig. 2:**
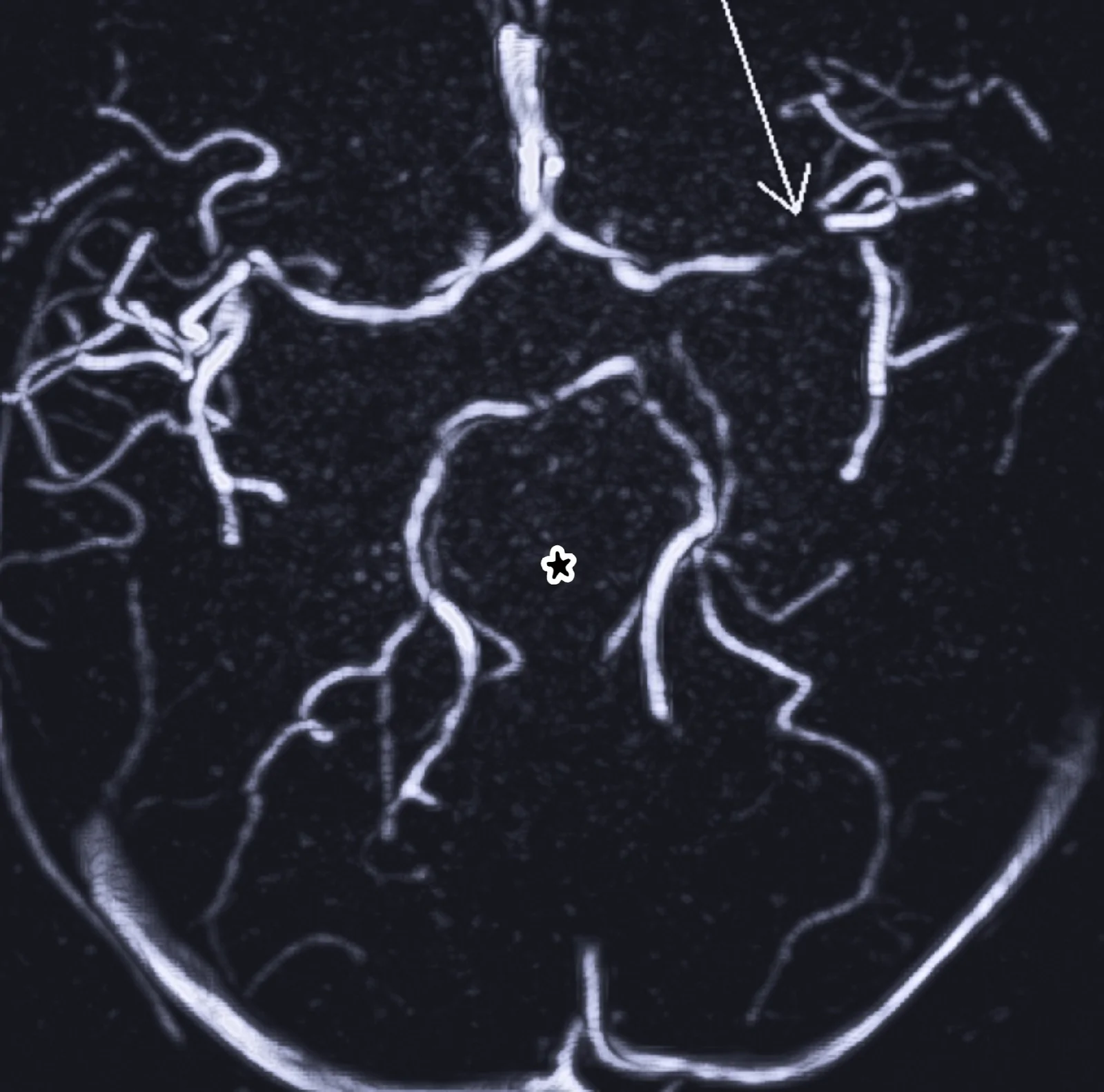
Intracranial time-of-flight MR angiography (3D-TOF MRA, axial maximum intensity projection). The white arrow indicates abrupt interruption of flow signal at the left M1 segment of the middle cerebral artery, consistent with thrombotic occlusion. The central asterisk (*) indicates complete absence of basilar artery flow signal throughout its entire course, consistent with thrombotic occlusion of the vertebrobasilar system. The remaining major intracranial arteries demonstrate preserved flow signal without additional significant stenoses, supporting the interpretation of focal thrombotic occlusions rather than diffuse vasculopathy.

**Chronic white matter changes:** Extensive confluent T2/FLAIR hyperintensities were present throughout the periventricular and deep white matter, corresponding to Fazekas grade 2 leukoaraiosis [4]. A focal T2/FLAIR hyperintensity was additionally identified in the genu of the corpus callosum, with signal characteristics consistent with chronic ischemic gliosis (no diffusion restriction, no gadolinium enhancement) (Fig. 1, Fig. 2C).

**Bilateral middle cerebellar peduncle (MCP) hyperintensities:** A salient finding was the presence of symmetric, confluent T2/FLAIR hyperintensities involving both MCPs, without associated diffusion restriction and without post-contrast enhancement (Fig. 1D). This pattern was interpreted as consistent with chronic ischemic gliosis rather than an acute ischemic event. A detailed etiological reasoning is given in the discussion.

### MR angiography (Fig. 2)

Time-of-flight MR angiography demonstrated an occlusive thrombus in the left M1 segment of the middle cerebral artery and a complete absence of flow signal in the basilar artery, consistent with subacute or chronic thrombosis. The remaining intracranial arteries showed no significant stenotic lesions, supporting the interpretation of focal thrombotic occlusions rather than diffuse vasculopathy.

### Cardiovascular and peripheral vascular work-up

**Fig. 3:**
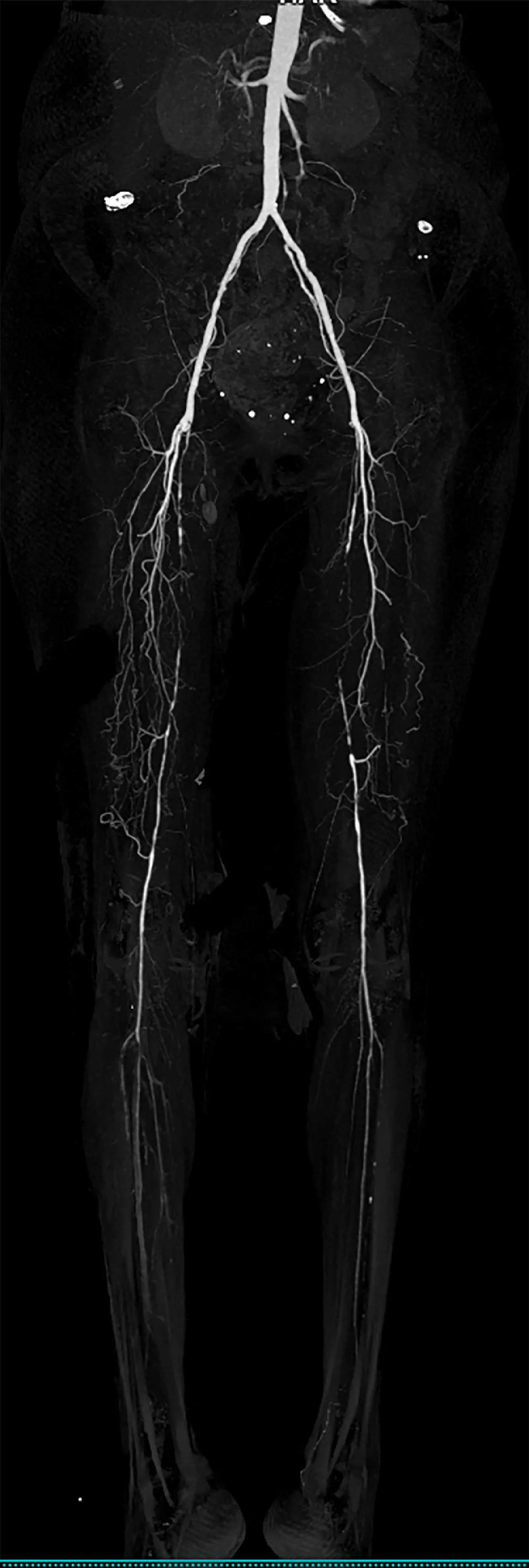
CT angiography of the lower extremities (maximum intensity projection, coronal view). Coronal MIP reconstruction of the aorto-iliac and lower extremity arterial tree demonstrates patent infrarenal aorta with abrupt loss of flow signal at the level of both common femoral arteries, consistent with bilateral femoral artery thrombotic occlusion. Reconstitution of the bilateral superficial femoral arteries is observed, likely via pelvic collateral pathways. Multisegmental irregularity and discontinuity of flow signal are noted along the superficial femoral, popliteal, and crural axes bilaterally, with more pronounced signal attenuation on the right side, consistent with critical limb ischemia. These findings confirm the diagnosis of severe multilevel lower extremity arterial disease (LEAD) in the setting of a systemic arterial thrombotic process.

Transthoracic echocardiography (TTE) revealed hypertensive cardiomyopathy without intracavitary thrombus, patent foramen ovale, or significant valvulopathy; no cardioembolic source was identified. Transesophageal echocardiography (TEE) was not performed owing to the patient's clinical instability at the time of cardiac evaluation. We discuss about the diagnostic implications this limitation in the corresponding chapter of the discussion. Carotid duplex showed only moderate atheromatous plaque without significant stenosis. CT angiography of the lower extremities (Fig. 3) confirmed bilateral femoral artery thrombosis with multilevel infrainguinal occlusions, more severe on the right, consistent with known LEAD.

### Laboratory investigations

Initial blood tests revealed a marked leukocytosis at 45,190 white blood cells per mm³ with a predominance of neutrophils (90.7%) associated with a high C-Reactive Protein (192 mg/L), indicating a significant inflammatory or infectious response. A normochromic normocytic anemia was noted, with a hemoglobin level of 8.1 g/dL. Renal function was mildly impaired. Hyperuricemia was present at 78 mg/L. Lipids, transaminases and electrolyte levels were normal. A targeted laboratory work-up including antinuclear antibodies (ANA), lupus anticoagulant, anticardiolipin antibodies (IgG/IgM), anti-β2-glycoprotein I antibodies, protein C and S activity, antithrombin III, Factor V Leiden mutation, prothrombin G20210A mutation, and homocysteine was requested as part of the etiological investigation but could not be performed before the patient's death.

### Clinical course and outcome

The patient was started on anticoagulation therapy and broad-spectrum antibiotics for the pre-existing lower limb infection. However, her limb ischemia progressed to critical limb ischemia despite medical management. Because revascularization was not feasible, a right above-knee amputation was performed. Postoperatively, she developed surgical site infection leading to sepsis and multiorgan failure, and died three weeks after admission.

## Discussion

### A severe systemic thrombotic process with diagnostic uncertainty

This case illustrates a catastrophic systemic arterial thrombotic process in a 50-year-old woman with hypertension and diabetes. Although these conventional vascular risk factors contribute to atherosclerosis, the clinical and imaging profile cannot be adequately explained by atherosclerotic disease or cardio-embolism alone: the absence of significant carotid or vertebral stenosis, the coexistence of simultaneous large-artery cerebral occlusions (left M1 and basilar artery) with bilateral femoral thrombosis, and the lack of an identified cardiac embolic source all argue against these mechanisms [1,2]. The marked leukocytosis (45,190/mm³) with neutrophilia raises the possibility of an underlying thrombo-inflammatory condition, such as antiphospholipid syndrome (APS), systemic vasculitis, or a reactive process secondary to extensive tissue ischemia [5,6].

APS is the leading hypothesis given the multiterritory arterial thromboses in a relatively young patient; however, it remains speculative in the absence of confirmatory antibody testing. Other diagnoses — hereditary thrombophilia, Behçet's disease, paraneoplastic coagulopathy — cannot be excluded [2,5]. Critically, the absence of procalcitonin measurement and blood cultures at admission precludes confirmation of an infectious origin of the right lower extremity tegumental lesions, which may have contributed to a pro-thrombotic state. This case underscores a key clinical lesson: in any patient with multiterritory arterial thromboses, antiphospholipid antibody testing and a complete thrombophilia screen should be initiated urgently and in parallel with imaging, before irreversible decisions — such as amputation — are made. Anticoagulation was empirically initiated and would have remained the treatment of choice had APS been confirmed, validating the clinical decision made in the absence of definitive etiological diagnosis [6].

The absence of a definitive etiological diagnosis in this case does not diminish its educational value. Fatal cases of suspected thromboinflammatory arteriopathy where workup could not be completed before death represent a recognized and reported clinical reality [2,5], and the radiological and clinical reasoning illustrated here retains full instructional value independently of a confirmed final diagnosis.

### Bilateral MCP T2/FLAIR hyperintensities: pathophysiology, differential diagnosis, and interpretation

The symmetric bilateral T2/FLAIR signal abnormality of the MCPs, without diffusion restriction or gadolinium enhancement, is a well-recognized but radiologically challenging pattern requiring systematic analysis [3,7]. It is essential to understand the pathophysiological mechanisms that may underlie this finding in order to avoid diagnostic error.

**Pathophysiological basis:** The middle cerebellar peduncles are the primary white matter tracts connecting the basis pontis to the cerebellar hemispheres via the transverse pontine fibers. Their vascular supply derives predominantly from the anterior inferior cerebellar artery (AICA) and long circumferential branches of the basilar artery. In the context of basilar artery occlusion, as documented in this case, chronic hypoperfusion of these territories leads to ischemic rarefaction and reactive gliosis — producing the T2/FLAIR hyperintensity pattern observed [3,8]. Wallerian degeneration may contribute as a secondary mechanism: the right paramedian pontine infarct could have triggered anterograde axonal degeneration along the corticopontocerebellar pathway toward the ipsilateral MCP, as described in the literature [7]. However, the bilateral and symmetric nature of the lesions makes this mechanism less plausible as the primary explanation. Both mechanisms are consistent with the absence of diffusion restriction and gadolinium enhancement, which exclude acute infarction and active demyelination respectively.

The differential diagnosis of bilateral MCP T2/FLAIR hyperintensities is broad and encompasses chronic ischemic gliosis, Wallerian degeneration, posterior reversible encephalopathy syndrome (PRES), multiple sclerosis and demyelinating disorders, CNS vasculitis, metabolic encephalopathies (Wernicke, osmotic demyelination, metachromatic leukodystrophy), and antiphospholipid syndrome or thrombotic microangiopathy. Table 1 provides a structured comparison of these etiologies with their key discriminating neuroimaging features and their applicability to the present case.

**Table 1:** Differential diagnosis of bilateral middle cerebellar peduncle (MCP) T2/FLAIR hyperintensities: neuroimaging features and applicability to the present case.

Etiology	MRI signal pattern	DWI restriction	Gd enhancement	Key discriminating features	Assessment in present case
**Chronic ischemic gliosis (vertebrobasilar hypoperfusion)**	Bilateral symmetric T2/FLAIR; may extend to pons and cerebellar hemispheres	Absent (chronic)	Absent	Basilar/vertebral occlusion or stenosis on MRA; diffuse leukoaraiosis; lesions stable on follow-up	CONSISTENT — basilar artery occlusion confirmed, Fazekas 2 leukoaraiosis, no DWI restriction, no enhancement
**Wallerian degeneration (secondary to pontine infarct)**	T2/FLAIR hyperintensity along cortico-ponto-cerebellar pathway; may be unilateral or bilateral	Absent (subacute-chronic)	Absent	Temporal relationship with ipsilateral pontine infarct (weeks to months); may be asymmetric	POSSIBLE — right paramedian pontine infarct present; MCP signal may partly reflect anterograde degeneration
**Posterior Reversible Encephalopathy Syndrome (PRES)**	Bilateral T2/FLAIR; parieto-occipital predominance; MCP involvement possible	Absent (vasogenic edema); present if infarction occurs	Variable, patchy	Acute hypertensive crisis or immunosuppression; cortical/subcortical involvement; full or partial reversibility on follow-up	UNLIKELY — no cortical edema, no enhancement, BP moderately elevated (no crisis), no follow-up to assess reversibility
**Multiple sclerosis/demyelinating disease**	Ovoid periventricular, juxtacortical, infratentorial plaques; MCP plaques possible (esp. neuromyelitis optica spectrum)	Present in active plaques	Nodular or ring-like in active plaques	Age <50, female predominance; relapsing-remitting history; CSF oligoclonal bands; dissemination in space and time (McDonald criteria)	UNLIKELY — no periventricular or juxtacortical plaques, no enhancement, no demyelinating clinical history
**CNS vasculitis**	Multifocal T2/FLAIR hyperintensities; any territory including posterior fossa; leptomeningeal enhancement possible	Present in ischemic territories	Leptomeningeal or vessel-wall enhancement (high-resolution vessel wall MRI)	Headache, CSF pleocytosis, elevated ESR/CRP; vessel wall MRI abnormalities; biopsy for confirmation	POSSIBLE but unconfirmed — no vessel wall MRI, no CSF, no enhancement; leukocytosis present but non-specific
**Metabolic encephalopathy (Wernicke, osmotic demyelination, metachromatic leukodystrophy)**	Symmetric T2/FLAIR in specific locations: periaqueductal/mammillary (Wernicke); central pons (ODS); diffuse white matter (MLD)	Variable	Variable	Specific metabolic context (thiamine deficiency, rapid sodium correction, lysosomal enzyme deficiency); no central pontine involvement here	UNLIKELY — no relevant metabolic history, normal electrolytes, no central pontine signal abnormality
**Antiphospholipid syndrome/thrombotic microangiopathy**	Multifocal white matter T2/FLAIR; ischemic infarcts in multiple vascular territories including posterior fossa	Present in acute/subacute ischemic lesions	Absent (ischemic mechanism)	Multiterritory arterial/venous thromboses; positive aPL antibodies (at least twice, 12 weeks apart); thrombocytopenia; peripheral thrombosis	POSSIBLE but unconfirmed — multiterritory occlusions and femoral thromboses present; aPL testing not completed before death

Abbreviations: aPL, antiphospholipid antibodies; BP, blood pressure; CNS, central nervous system; CSF, cerebrospinal fluid; DWI, diffusion-weighted imaging; ESR, erythrocyte sedimentation rate; Gd, gadolinium; MCP, middle cerebellar peduncle; MLD, metachromatic leukodystrophy; MRA, MR angiography; ODS, osmotic demyelination syndrome; PRES, posterior reversible encephalopathy syndrome.

In the present case, the combination of documented basilar artery occlusion on TOF-MRA, Fazekas grade 2 leukoaraiosis reflecting longstanding cerebrovascular disease, chronic gliotic lesion in the corpus callosum genu, and absence of any clinical or biological features of demyelination, metabolic disease, or hypertensive crisis makes chronic ischemic gliosis the most parsimonious interpretation. PRES is less likely given the absence of enhancement, bilateral symmetry without cortical involvement, and the absence of an acute hypertensive crisis at MRI time. This interpretation must remain provisional, however, given the single MRI time-point at seven days post-ictus and the absence of follow-up imaging.

### Limitations

This report has five principal limitations. (1) Incomplete etiological work-up: antiphospholipid antibodies and thrombophilia screening could not be performed before death. (2) Absence of key infectious markers: procalcitonin and blood cultures were not obtained at admission, precluding formal confirmation of bacteremia contributing to the prothrombotic state. (3) Single MRI time-point: lack of follow-up imaging prevents definitive characterization of the MCP lesions as chronic. (4) No autopsy: the histopathological nature of the arterial thromboses and the exact cause of death remain unknown. (5) TEE was not performed, although it would have allowed more sensitive detection of a patent foramen ovale (PFO), left ventricular thrombus, and aortic arch atheromatous plaques — all potential embolic sources not fully excluded by TTE alone [9]. TEE was clinically unreasonable in this hemodynamically compromised, septic patient. Furthermore, TEE carries well-documented limitations in PFO workup: its sensitivity is approximately 89% when validated against autopsy findings [10], and sedation during the procedure frequently compromises the patient's ability to perform an adequate Valsalva maneuver, leading to false-negative results [10,11]. Irrespective of these limitations, the multiterritory arterial thrombotic pattern — simultaneously affecting intracranial and bilateral femoral territories — is more consistent with a systemic prothrombotic process than with paradoxical embolism via PFO, making the latter an unlikely dominant mechanism.

Despite these limitations, the combination of multiterritory acute infarcts, chronic white matter changes, bilateral MCP T2/FLAIR hyperintensities, and peripheral arterial thromboses is rare and has important clinical implications.

## Conclusion

This case report conveys two radiologically and clinically actionable teaching points. First, bilateral MCP T2/FLAIR hyperintensities without diffusion restriction or gadolinium enhancement, occurring in the setting of documented basilar artery occlusion and longstanding leukoaraiosis, should be interpreted as chronic ischemic gliosis — a conclusion supported by systematic differential diagnosis and a structured review of discriminating neuroimaging features. Second, in any patient presenting with multiterritory arterial thrombosis concurrent with active systemic infection, antiphospholipid antibody testing and a complete thrombophilia screen must be initiated urgently and in parallel with imaging, before irreversible clinical decisions are made. The catastrophic outcome of this case — death within three weeks from multiorgan failure — underscores the fatal potential of unrecognized thromboinflammatory arteriopathy and the absolute priority of a systematic etiological approach, even in resource-limited settings.

## Ethics statement

This case report was approved by the Ethics Committee of the Institut de Cardiologie d’Abidjan (ICA), Abidjan, Côte d’Ivoire.

## Patient consent

Written informed consent was obtained from the patient’s next of kin for the publication of this case report and accompanying images, following the patient’s death.

## Declaration of generative AI and AI-assisted technologies in the writing process

The authors used an AI-assisted writing tool (Claude, Anthropic) for language editing and manuscript structuring. All clinical content, data interpretation, and intellectual contributions are solely those of the authors.

## Appendix Supplementary materials

Image, application 1
