# Supplementary material for: Multiterritory Acute Ischemic Stroke with Bilateral Middle Cerebellar Peduncle T2/FLAIR Hyperintensities in the Setting of a Fatal Systemic Arterial Thrombotic Process: A Case Report
Source: Radiol Case Rep. 2026 Sep 15;21(12):6124–30. doi: 10.1016/j.radcr.2026.08.046 (PMC13598526; doi:10.1016/j.radcr.2026.08.046)
Supplement: Supplementary file 1 [file mmc1.docx]

**CARE Checklist**

*Case Reports in Neurology (Karger) — Mandatory Submission Document*

Manuscript title: Multiterritory Acute Ischemic Stroke with Bilateral Middle Cerebellar Peduncle T2/FLAIR Hyperintensities Revealing a Fatal Systemic Arteriopathy: A Case Report

| **Item** | **Topic** | **Sub-item checklist** | **Location in manuscript** |
| --- | --- | --- | --- |
| **1** | **Title** | The words case-report should appear in the title | Title page — present in title |
| **2** | **Key words** | 2 to 5 keywords identifying diagnoses or interventions, including case-report | Abstract — 7 keywords provided |
| **3a** | **Abstract — Introduction** | What is unique about this case? What does it add to the medical literature? | Abstract, Background |
| **3b** | **Abstract — Main symptoms and/or important clinical findings** | State main symptom(s) and key finding(s) | Abstract, Case Presentation |
| **3c** | **Abstract — The main diagnoses, therapeutic interventions, and outcomes** | State main diagnoses, key interventions, and outcomes | Abstract, Case Presentation + Conclusion |
| **3d** | **Abstract — Conclusion** | What is the main take-away lesson from this case? | Abstract, Conclusion |
| **4** | **Introduction** | Briefly summarize why this case is unique, referencing the medical literature | Introduction, paragraphs 1–3 |
| **5a** | **Patient information — De-identified patient specific information** | Age, sex, relevant demographics; chief complaint(s); medical, family, and psychosocial history; relevant comorbidities | Case Presentation, Clinical History section |
| **5b** | **Patient information — Primary concerns and symptoms** | Main symptoms at presentation | Case Presentation, Clinical History section |
| **5c** | **Patient information — Medical, family, and psychosocial history** | Relevant history including interventions and relevant family and genetic information | Case Presentation, Clinical History section |
| **5d** | **Patient information — Relevant past interventions and their outcomes** | N/A — no relevant prior interventions in this case (stated in text) | Case Presentation; limitation acknowledged in Discussion |
| **6a** | **Clinical findings — Physical examination findings** | Neurological examination findings, NIHSS score, BP | Case Presentation, Clinical History section |
| **6b** | **Clinical findings — Important findings from other diagnostic tests** | MRI, MRA, echocardiography, CTA, blood tests | Case Presentation, all imaging and laboratory subsections |
| **7a** | **Timeline — Important dates and times in this case** | Symptom onset (1 week prior), admission, amputation, death (3 weeks post-admission) | Case Presentation; noted throughout |
| **7b** | **Timeline — Summary of information by date/time** | Clinical timeline integrated into case narrative | Case Presentation (sequential subsections) |
| **8a** | **Diagnostic assessment — Diagnostic methods** | MRI brain (DWI, FLAIR, T1±Gd, TOF-MRA), echocardiography, duplex ultrasound, CTA lower limbs, blood tests | Case Presentation |
| **8b** | **Diagnostic assessment — Diagnostic challenges** | Incomplete thrombophilia work-up, no infectious markers, single MRI, no autopsy | Discussion, Limitations subsection |
| **8c** | **Diagnostic assessment — Diagnostic reasoning** | Exclusion of cardio-embolism and atherosclerosis; discussion of APS and other thrombo-inflammatory causes | Discussion, Systemic Thrombotic Process subsection |
| **8d** | **Diagnostic assessment — Prognostic characteristics where applicable** | Catastrophic multiterritory occlusions with fatal outcome | Discussion + Conclusion |
| **9a** | **Therapeutic intervention — Types of therapeutic intervention** | Antiplatelet therapy (aspirin 100 mg/day); right above-knee amputation | Case Presentation, Clinical Course subsection |
| **9b** | **Therapeutic intervention — Administration of therapeutic intervention** | Aspirin initiated at admission; amputation performed for critical limb ischemia | Case Presentation, Clinical Course subsection |
| **9c** | **Therapeutic intervention — Changes in therapeutic intervention with reasons for changes** | Surgical revascularization not feasible; escalation to amputation | Case Presentation, Clinical Course subsection |
| **10a** | **Follow-up and outcomes — Clinician and patient-assessed outcomes** | Death at 3 weeks from sepsis and multiorgan failure post-amputation | Case Presentation, Clinical Course subsection |
| **10b** | **Follow-up and outcomes — Important follow-up diagnostic and other test results** | No follow-up MRI obtained; thrombophilia screen incomplete; no autopsy | Discussion, Limitations subsection |
| **10c** | **Follow-up and outcomes — Intervention adherence and tolerability** | Patient died before further interventions; aspirin tolerability not assessable | Case Presentation |
| **11** | **Discussion — Strengths and limitations of case management** | Comprehensive MRI protocol (strength); incomplete etiological work-up, no infectious markers, single MRI, no autopsy (limitations) | Discussion, Limitations subsection |
| **12** | **Patient perspective** | Patient perspective not obtainable; consent obtained from next of kin | Statement of Ethics |
| **13** | **Informed consent** | Written informed consent obtained from patient's next of kin; available for review by Editor | Statement of Ethics |

*Reference: Riley DS, Barber MS, Kienle GS, et al. CARE guidelines for case reports: explanation and elaboration document. J Clin Epidemiol. 2017;89:218–235.*
